# Translation and validation of a German version of the “Hopkins Competency Assessment Test,” a brief method for evaluating patients’ capacity to give consent

**DOI:** 10.1186/s12888-026-08401-6

**Published:** 2026-07-24

**Authors:** Hannah C. Keppeler, Mounira Jabat, Lena Machetanz, Mona Redlich Bossy, Achim Burrer, Stefan Vetter, Erich Seifritz, Johannes Kirchebner, Stephan T. Egger

**Affiliations:** 1 University of Zurich, Zurich, Switzerland; 2 Psychiatric University Hospital Zurich, Zurich, Switzerland; 3 Penitentiary Services of the Canton of Zurich, Zurich, Switzerland; 4 University Psychiatric Centre Bern, Bern, Switzerland

**Keywords:** Competency assessment, Psychiatric patients, Clinical trial, Participation

## Abstract

**Background:**

The “Hopkins Competency Assessment Test” (HCAT) assesses a patient’s ability to provide written informed consent or advance directives. It includes an essay for three reading difficulty levels (12th, 8th, and 6th school grade) and an understanding questionnaire. Currently, there is no equivalent test to the HCAT in German. Therefore, we translated, adapted, and evaluated a German version of the HCAT.

**Methods:**

We conducted a cross-sectional study to evaluate the psychometric properties of the HCAT in a clinical and a non-clinical sample. Statistical analysis included ANOVA and t-tests for group comparisons. Cronbach’s alpha was used to assess internal consistency; an exploratory factor analysis (EFA) was conducted to examine the underlying factor structure; and network analysis with centrality indices to explore the relational structure among HCAT items and to identify particularly central components within the network. The data collection and analysis were approved (BASEC 2021-01123) by the ethics committee of the Canton of Zurich.

**Results:**

We enrolled two samples: one clinical, consisting of 110 psychiatric inpatients, and for comparison, one non-clinical, consisting of 32 participants recruited from hospital staff. We present the results from both samples, unless stated otherwise. The total number of participants, both clinical and non-clinical, was 142 individuals; the majority were male (n = 117, 82.4%), with a mean age of 37.5 years. Most had no education beyond regular school (n = 69, 48.6%). Among the psychiatric patients, schizophrenia (n = 87, 75.5%) was the primary diagnosis, followed by affective disorders (24.5%). Patients often had comorbid substance use disorders (n = 39, 35.5%). A considerable proportion completed the HCAT successfully in the first attempt (n = 100, 70.4%) or the second attempt (n = 20, 14.1%). All participants of the non-clinical sample completed the HCAT successfully on the first attempt. For those requiring more than one attempt, the Intraclass Correlation Coefficient between the different HCAT reading difficulty versions was 0.07 (F(19,6.81) = 1.75, p = .23). The essay took 2.0 (0.8) minutes to read and 5.3 (3.1) minutes to answer. Persons in psychiatric treatment required more time, more attempts, and had lower scores. The Cronbach’s alpha was 0.88. The CFA suggested a two-factor model as the preferred solution. In our interpretation, the network analysis revealed the dynamics influencing consent capacity and highlighted the role of risk-benefit valuation for patients (or potential participants) in informed decision-making.

**Conclusion:**

The German version of the HCAT demonstrated promising psychometric properties and may be a useful tool for screening for decisional capacity in psychiatric patients.

**Supplementary Information:**

The online version contains supplementary material available at https://doi.org/10.1186/s12888-026-08401-6.

## Background

Written informed consent implies voluntariness and understanding of the proposed treatment, including its risks, benefits, and alternatives. Generally, in adults, the ability to give voluntary informed consent is assumed [5]. However, this presumption relies on specific legal assumptions and clinical criteria, including the appreciation of the current situation and its consequences, the ability to understand relevant information, appreciate the situation, reason about treatment options and their consequences, the rational manipulation of information, and the ability to communicate a choice [5].

Decisional capacity, however, is not a static construct but a context-dependent and potentially fluctuating condition. It can be influenced by distress, haste, availability, situation, and the clinical setting [8]. Furthermore, the reliance on unstructured clinical judgment alone may be insufficient to ensure transparency and consistency [2]. Thus, in psychiatric conditions, the exploration of capacity should include psychodynamic aspects, the stability of the patient’s mental status over time, and the effect of the setting in which consent is obtained [3, 6, 8].

Assessing a patient’s capacity to consent is a dynamic, continuous process that is often challenging [24]. Therefore, capacity assessments should be proportionate, context-sensitive, and primarily aimed at identifying situations in which a comprehensive evaluation is warranted [3]. Structural approaches to capacity assessment aim to support clinical decision-making while avoiding undue paternalism [37]. Beyond issues of availability, the reliability and validity of currently used instruments for assessing decisional capacity remain subjects of ongoing discussion; without a universally validated standard across clinical contexts [4, 28, 31]. Thus, screening instruments for impaired capacity facilitate the identification of patients who may require a more detailed evaluation, rather than replacing clinical judgment altogether [3, 4].

In this context, the “Hopkins Competency Assessment Test” was developed as a brief instrument to evaluate a patient’s competency to provide written informed consent or to complete advance directives, to support a clinician in forming an opinion [22, 23]. It consists of a short essay on informed consent and durable power of attorney, followed by a questionnaire to assess patients’ understanding of the essay. The essay has three versions with different reading difficulty levels. If the patient scores low in the questionnaire, an easier version of the essay is presented. Since its first publication, the Hopkins Competency Assessment has proven to be an easy-to-use and valid tool for screening the competency of patients with a psychiatric disorder [28, 39, 46]. To the best of our knowledge, there is no equivalent test to the Hopkins Competency Assessment Test in German. Therefore, we translated the original English version, “Hopkins Competency Assessment Test,” into German.

The present study aims to evaluate the administration and feasibility of the German version of the HCAT and to analyze its psychometric properties in a clinical and non-clinical population. We expect staff and patients to participate in and complete the HCAT procedures. We hypothesized that the use of the HCAT would be brief and able to differentiate between clinical and non-clinical populations. As in the original English version, we expected the German version of the HCAT to demonstrate good psychometric properties. By exploring the HCAT structure, we expect to gain insight into the mechanisms underlying consent capacity.

## Methods

### Study design and sample population

Study participants were recruited from adult patients with schizophrenia spectrum disorder currently hospitalized at the University Hospital of Psychiatry Zurich. Patients were hospitalized for psychiatric treatment either in a forensic facility (for court-mandated treatment) or voluntarily in a regular psychiatric ward. To minimize selection bias, we enrolled all patients who were able and willing to collaborate. To ensure transparency and avoid misuse, the assessment (and documentation) of capacity to consent, using a screening instrument, was required as a first step before providing proper information and potential enrollment in a clinical trial. Capacity to consent was clinically examined by the case-managing clinical staff, who was either a licensed psychiatrist or psychologist. If patients were under guardianship (according to Articles 16 and 17 of the ZGB), the legal guardian (or tutor) was approached and involved in the decision-making process. The exclusion criteria were patients with acute psychotic symptoms, non-German speakers, and those who rejected participation. The HCAT capacity assessment was conducted within the context of screening procedures for participation in a superordinate pharmacological study. The study was designed in accordance with the Declaration of Helsinki and approved by the ethics committee of the Canton of Zurich, Switzerland (BASEC 2021 − 01123). Clinical trial number: not applicable. The assessment should be carried out by independent staff who are neither involved in treatment nor in the main clinical trial. We included a second sample to ensure a non- clinical comparison group. Therefore, we recruited a convenience sample of all professional groups working at the hospital at that time (nurses, social workers, administrative staff, cleaners). Informed consent to participate was obtained from all participants.

### Demographic and clinical characteristics of the sample

We collected age, sex, civil status, highest completed education, and legal-contractual capacity of all inpatients at that time who were willing to participate. Data sources included personal details provided by patients, relatives, and guardians, as well as medical records. We further recorded the main diagnosis, comorbid disorders, and duration of illness. We used the Health of the Nation Outcome Scale (HoNOS) score at admission to assess severity and need for care [15, 21, 47]. The HoNOS assess 12 different domains covering “behaviour” (aggressiveness; non-accidental self-injury; problem drinking or drug taking), “impairment” (cognitive problems; physical illness or disability problems), “symptoms” (hallucinations and delusions; depressed mood; other mental and behavioural problems) and “social problems” (problems with relationships; problems with activities of daily living; problems with living conditions; problems with occupation and activities). Each item is rated on a five-point Likert scale from 0 (“no problem”) to 4 (“severe to very severe problem”). The HoNOS ranges from 0 to 48, with higher scores indicating a more severe disorder and a greater need for care [15].

### The Hopkins Competency Assessment Test (HCAT)

The HCAT is a screening instrument designed to evaluate the capacity to provide informed consent. It is delivered in two steps. First, a short informational essay outlining the principles of informed consent and durable power of attorney, and second, a ten-item questionnaire assessing comprehension of the reading material. To accommodate varying reading abilities, the essay is available in three versions of decreasing complexity, corresponding to U.S. 12th-grade, 8th-grade, and 6th-grade reading levels. Participants initially received the 12th-grade version. In cases of difficulty or insufficient performance, simplified versions were presented sequentially; the questionnaire was presented unaltered [22, 23].

The HCAT questionnaire assesses understanding of the information presented in the essay. Its items reflect key elements of decisional capacity and informed consent, including understanding the proposed procedure, expected benefits, possible risks, and alternatives, and the ability to decide for or against treatment. Additional questions address situations in which decision-making or communication may deteriorate and assess whether patients understand the role of a representative, treatment stipulations, and advance directives. Thus, the questionnaire covers several core components of consent-related decision-making [22, 23]. For further details, see Table 3 and Appendix 1.

### Translation process to a German version of the HCAT

The translation of the Hopkins Competency Assessment Test from English to German was conducted in accordance with current standards for the translation of health measurement instruments [38]. First, the instrument was translated from English to German by a native German speaker. The fidelity of the translated version to the original English version was discussed and modified accordingly. An expert adapted the complexity of the essay to fulfill the reading and comprehension abilities of the sixth, eighth, and twelfth grades. This version was translated back to English by a native English speaker. The authors were given the German version and the English retranslation for approval. The authors of the original English version of the HCAT were not involved in this process; our version of the HCAT is not an official translation. The German version used in this study is available in Appendix 1.

### Outcome and procedure

The administration of the HCAT followed the same order as in the original English version [22, 23]. Each participant was asked to read the 12th -grade version of the HCAT; afterward, the examiner played an audio version of the material to eliminate illiteracy as a confounding factor. If the participant scored eight or higher on answering the questions, the examination was finished, and the score was recorded. If the score was seven or less, the examiner and participant went through the 8th -grade version as they did with the 12th -grade version, and the questionnaire was administered again afterward. If the subject scored seven or less, the questionnaire was administered after reading and hearing the 6th -grade version. For each attempt, we recorded the time spent reading the essay and answering the questionnaire. In a specific workshop, the recruiting personnel learned and practiced the sequence and handling of the HCAT. Afterward, they administered the HCAT to the participants of both samples.

Administration and feasibility were evaluated using several descriptive measures, including reading time, response time, successful completion rates, and the number of attempts required to complete the HCAT at different reading levels. In addition, we assessed whether participants could complete the assessment after the first, second, or third administration. Furthermore, we consider that participant retention through the screening process (even after failing to achieve a sufficient score) is indicative of the HCAT’s feasibility and applicability [43]. In contrast to the original and previous HCAT studies [22, 23, 46], participants in the present study did not read and listen to the essay simultaneously. Instead, the essay was first presented in written form and subsequently read aloud. This procedure was chosen to reflect clinical practice better and to allow separate assessment of reading and listening comprehension.

### Statistical analysis

We used descriptive statistics (means, standard deviations, and percentages) to represent our sample’s demographic and clinical characteristics. For the statistical analysis of group differences, we used analysis of variance (ANOVA) and Student’s t-test for pairwise comparisons. Missing values were not imputed. We corrected for multiple comparisons using the Bonferroni method, and we set the significance threshold at *p* < .01.

We evaluated the time required to read the essay and to answer the questionnaire. We calculated a reading index based on the text’s complexity and the time required to read it [26]. We also calculated a performance index, based on the time required to answer and the number of correct answers. Finally, for those participants who required more than one attempt and completed two (i.e., 12th and 8th) or three (i.e., 12th, 8th, and 6th) forms of the HCAT, we calculated the cumulative time spent reading the essays and answering the questionnaire. We calculated the intraclass correlation coefficient (ICC) for each item to assess whether the types of answers were consistent across the different versions.

We evaluated the assumptions for the exploratory factor analysis (EFA). The internal consistency of the HCAT was determined using Cronbach’s alpha and Donald’s omega [48]. The Kaiser- Meyer- Olkin for sampling adequacy; the Bartlett’s test for sphericity; and the eigenvalues represented as a screen plot. We use factor loadings to indicate how strongly an item is associated with an underlying factor in the HCAT; we consider loadings < 0.30 as weak, 0.30–0.50 as moderate, 0.50–0.70 as strong, and > 0.70 as very strong.

For the graphical representation of the HCAT, we calculated a variation-correlation matrix. We subsequently performed a network analysis to identify not only the structure but also the dynamics and relationships among the individual elements of the HCAT. We performed a confirmatory factor analysis (CFA)for the validation and selection of the model, we used several commonly used indices to assess the goodness of fit: chi-square, the comparative fit index (CFI), the Tucker‒Lewis index (TLI), the Akaike information criterion (AIC), the Bayesian information criterion (BIC), and the standardized root mean square residual (SRMR) [13, 20].

In network models, variables (i.e., items) are represented as nodes connected by edges, which correspond to undirected regularized partial correlations [9]. The network model of the HCAT was calculated using the “extended Bayesian information criterion” (EBIC) and the “least absolute shrinkage and selection operator” (LASSO) regularization methods implemented within a single Gaussian random field network. To control for the degree of shrinkage, we used a low hyperparameter (gamma = 0.0) to maximize network stability and balance sensitivity and specificity [16].

To describe the network’s topological properties, we used network centrality measures. (strength, closeness, betweenness, and expected influence). The node strength is the sum of the absolute edge weights per node. Node closeness quantifies the distance between a node and all other nodes by averaging the shortest paths to them. The node betweenness quantifies how often a node lies on the shortest path connecting two other nodes. The sign of the edge weight determines the node’s expected influence; it quantifies how much variance the node’s neighbors explain. To test the accuracy and stability of the network parameters, we estimated confidence intervals for the edge weights and the correlation stability coefficient using nonparametric bootstrapping [17].

For the statistical analyses and figures, we used RStudio (2026.04.0 + 526), the statistical software R (4.6.0), and the R packages *tidyverse* (2.0.0), *qgraph* (1.9.8), *bootnet* (1.8), and *networktools* (1.6.0).

## Results

### Demographic and clinical characteristics of the sample population

A total of 142 individuals participated in the current study. We included 78 patients receiving forensic treatment (in the high- and low-security wards), 32 patients receiving regular psychiatric treatment, and 32 non-clinical participants. The patients had a mean age of 37.5 (11.8) years. The majority were men (*n* = 117, 82.4%). Approximately half had not completed further education than regular school (*n* = 69, 48.6%). Among patients (*n* = 110), the most common diagnosis was schizophrenia (*n* = 83, 75.5%), followed by an affective disorder (*n* = 27, 24.5%), with approximately one-third of patients (*n* = 39, 35.5%) showing a comorbid substance use disorder. For further details, see Table 1.

**Table 1:** Demographic and clinical characteristics of the sample population

	Total	Controls	Regular	Forensic		Statistic	*p*
				Low Security	High Security		
	*n* = 142	*n* = 32	*n* = 32	*n* = 59	*n* = 22		
**Demographic Variables**							
	*Mean (SD)*	*Mean (SD)*	*Mean (SD)*	*Mean (SD)*	*Mean (SD)*		
Age (years)	37.5 (11.8)	36.4 (13.0)	41.1 (11.4)	36.4 (11.1)	37.1 (12.6)	F(3,138) = 1.155	0.32
	*n (%)*	*n (%)*	*n (%)*	*n (%)*	*n (%)*		
Sex							
Male	117 (82.4)	24 (75.0)	23 (74.2)	49 (87.5)	19 (90.5)	χ² (3,142) = 4.425	0.22
Female	25 (17.6)	8 (25.0)	9 (25.8)	7 (12.5)	3 (9.5)		
Education							
Regular (or lower)	69 (48.6)	12 (37.5)	11 (35.5)	31 (57.4)	13 (61.9)	χ² (3,142) = 6.063	0.11
Apprenticeship (or higher)	73 (51.4)	20 (62.5)	21 (64.5)	25 (42,6)	9 (38.1)		
**Clinical Variables**							
Diagnosis							
Psychotic Disorder	83 (58.5)	n.ap.	20 (62.5)	47 (83.9)	16 (76.2)	χ² (2,110) = 5.159	0.07
Affective Disorder	27 (19.0)	n.ap.	12 (37.5)	9 (16.1)	6 (23.8)		
Legal Guardian (Tutor)	38 (26.8)	n.ap.	6 (19.4)	23 (42.6)	9 (42.9)	χ² (2,110) = 4.979	0.08
	*Mean (SD)*	*Mean (SD)*	*Mean (SD)*	*Mean (SD)*	*Mean (SD)*		
Duration of Illness (years)		n.ap.	11.4 (6.8)	11.8 (9.0)	10.2 (6.8)	F(2,107) = 0.342	0.71
HoNOS		n.ap.	26.7 (5.5)	21.4 (7.1)^a^ *	24.1 (8.7)	F(2,107) = 5.943	0.004

^a^ low security < high security = regular

* *p*<.01; ** *p*<.001

n.ap.: not applicable

Legend: demographic and clinical (when pertinent) characteristics of the samples. Regular education refers to completion of compulsory general schooling without additional vocational or academic qualifications. Values are presented as mean (standard deviation) or number (percentage), as appropriate

### Administration and feasibility of the German translation of the HCAT

All participants who initiated the screening procedure completed it, without any dropouts. One hundred participants (70.4%) completed the HCAT after the first attempt (i.e., 12th grade version), 20 participants (14.1%)after the second attempt (i.e., 8th grade version), and only six participants after a third attempt (i.e., 6th grade version). Sixteen participants (11.3%) failed to complete the HCAT after three attempts. The rate of unsuccessful HCAT completion was higher among those who needed a third attempt (χ²(2, 206) = 19.007; *p*<.001).

Participants required 2.0 (0.8) minutes to read the HCAT essay and 5.3 (3.1) minutes to answer the HCAT questionnaire. Those who completed the HCAT after the first attempt (i.e., the 12th -grade version) required 1.7 (0.8) minutes to read and 5.0 (2.8) minutes to complete the questionnaire. For the 8th grade version (i.e., the second round), participants required 2.1 (0. 5) minutes to read and 5.9 (4.1) minutes to answer the questionnaire. For the 6th -grade version (i.e., the third round), participants required 2.0 (0.6) minutes to read and 5.7 (2.9) minutes to answer the questionnaire. Participants read the 12th -grade12th-grade version faster than the 8th - and 6th -grade6th-grade versions (F(2,203) = 8.107; *p*<.001). For further details, see Table 2.

There was no difference in time to complete the HCAT questionnaire (*p*=.45); the 12th -grade version achieved a higher score than the 8th -grade version, and the 8th -grade version achieved a higher score than the 6th -grade version (F(2,203) = 18.966; *p*<.001). However, if the time required to complete the questionnaire was considered, the 12^th−^ and 8th -grade versions required similar time, whereas the 6th -grade version took significantly longer (F(2,203) = 5.15; *p*=.007). Furthermore, the 6th -grade version had the highest proportion of participants who scored low on the questionnaire (χ² (2,206) = 19.007; *p*<.001). For further details, see Table 2.

There were no differences between patients with one, two, or three attempts regarding their basic demographic characteristics: age (*p*=.43), sex (*p*=.27), and education (*p*=.20). Healthy controls required just one attempt to complete the HCAT questionnaire, with all of them achieving the total score (i.e., ten out of ten points). The clinical sample had more difficulties completing the HCAT, had a longer time to read (F(3,138) = 12.149; *p*<.001), and had a longer time to answer (F(3,138) = 12.341; *p*<.001), while they achieved a lower score (F(3,138) = 17.591; *p*<.001). Among those receiving treatment, we did not observe clinical differences regarding diagnosis (*p*=.06), comorbidity (*p*=.07), duration of illness (*p*=.83), or severity (as indicated by the HoNOS) (*p*=.65). There were also no differences in their legal capacity, as indicated by the presence of a legal guardian or tutor (*p* = .31).

**Table 2:** Results for the HCAT questionnaire according to the difficulty level of the HCAT essay

	*12th Grade*	*8th Grade*	*6th grade*	Statistic	*p*
	*n* = 142	*n* = 42	*n* = 22		
	Mean (SD)	Mean (SD)	Mean (SD)		
HCAT Essay					
Reading time (minutes)	*1.9 (0.8)* ^*a*^	*2.3 (0.7)*	2.3 (0.7)*	*F(2*,*203) = 8.107*	*< 0.001*
Reading Index	*3.0 (1.3)* ^*b*^ **	*5.3 (1.7)* ^*b*^ **	*7.4 (2.3)* ^*b*^ **	*F(2*,*203) = 102.454*	*< 0.001*
HCAT Questionnaire					
Answer Time (minutes)	*5.3 (3.1)*	*5.7 (3.6)*	*4.6 (2.5)*	*F(2*,*203) = 0.793*	*0.45*
Score (0 to 10)	*7.9 (2.8)* ^*a*^	5.9 (3.1) **	4.4 (3.0) **	*F(2*,*203) = 18.966*	*< 0.001*
Performance Index	*2.0 (1.4)*	*1.5 (1.4)*	*1.2 (1.0)* ^*c*^	*F(2*,*203) = 5.15*	*0.007*
	n (%)	n (%)	n (%)		
*Sufficient Score*	*100 (70.4%)*	*20 (47.6)*	*6 (27.3)* ^*c*^ **	*χ²(2*,*206) = 19.007*	*< 0.001*

^a^ 12th Grade < 8th Grade* = 6th Grade

^b^ 12th Grade < 8th Grade < 6th Grade

^c^ 12th Grade = 8th Grade > 6th Grade

* <0.01; ** <0.001

Legend: The reading index was calculated based on the text’s complexity and the time required to read, the performance index was calculated based on the time required to answer, and the number of correct answers

## a

For those completing the 12th and 8th grade questionnaires, the intraclass correlation coefficient was 0.07 (F(19,6.81) = 1.75, *p* = .23); for those completing the 12th, 8th, and 6th grade questionnaires, the intraclass correlation coefficient was 0.76 (F(21,17.5) = 14.4, *p* < .001).

### Psychometric properties and structure of the German HCAT

**Fig. 1:**
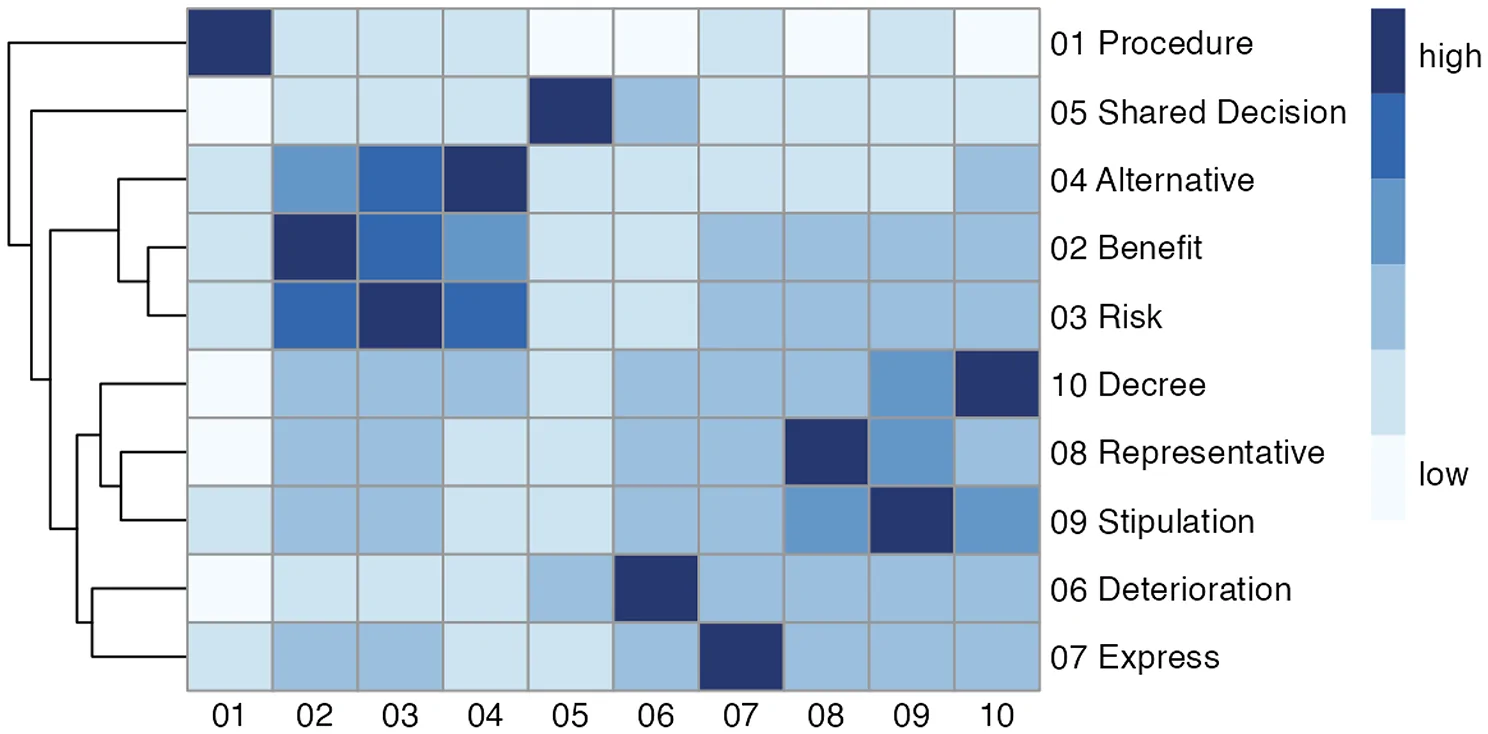
Variance‒covariance matrix of the HCAT-questionnaire

For the psychometric properties of the HCAT, all complete questionnaires (excluding those with reading difficulty) were included, yielding a total of 206. The HCAT yielded good internal consistency, with Cronbach’s alpha of 0.88 and a McDonald’s omega of 0.91. The Kaiser–Meyer–Olkin factor was 0.87, with an MSA ranging from 0.80 to 0.92 for each item. Both the KMO and the Measure of Sampling Adequacy (MSA) indicate adequate sampling, and the requirements for factorial analysis are met. The screen plot of the eigenvalues showed that a three-cluster model explained 77.5% of the variance, with two- and one-cluster models explaining 57.9% and 31.3%, respectively. Figure 1 shows the variation-correlation matrix, with correlations ranging from low (0.0 to 0.2) to high (0.8 to 1.0).

In the two-factor model, the first factor was composed of the procedure, including the benefits, risks, and alternatives; the second factor included elements of shared decision-making, including aspects when making or communicating a decision is not possible; and the surrogate decisions, such as a representative, stipulation, or even a patient decree, were included. In the three-factor model, the first factor consisted of the procedure and shared decision; the second factor consisted of the benefits, risks, and alternatives; the third and last factors included aspects when making or communicating a decision that is not possible; and the surrogate decisions, such as a representative, a stipulation, or even a patient decree. For further details, see Table 3.

**Table 3:** HCAT items (Questions) and results of the exploratory factorial analysis of the HCAT-questionnaire, factor loadings for each question of the HCAT

*Item*	*One Factor*	*Two Factor Model*		*Three Factor Model*		
	*Factor 01*	*Factor 01*	*Factor 02*	*Factor 01*	*Factor 02*	*Factor 03*
01 Procedure	What is the doctor going to do?					
	*0.43*	*0.46*	*-*	*0.72*	*-*	*-*
02 Benefit	What could go right?					
	*0.79*	*0.86*	*-*	*-*	*0.85*	*-*
03 Risk	What could go wrong?					
	*0.82*	*0.89*	*-*	*-*	*0.90*	*-*
04 Alternative	What else could the doctor do instead?					
	*0.74*	*0.92*	*-*	*-*	*0.94*	*-*
05 Shared Decision	After learning about the procedure, the patient can decide not to have the procedure done.					
	*0.53*	*-*	*0.48*	*-0.57*	*-*	*-*
06 Deterioration	After a while, the patient’s thinking may not be as good as it is now.					
	*0.68*	*-*	*0.80*	*-*	*-*	*0.75*
07 Express	Tell the doctor what the patient wants done.					
	*0.69*	*-*	*0.81*	*-*	*-*	*0.83*
08 Representative	Patients can write down who else the doctor can talk to in order to make medical decisions for them.					
	*0.74*	*-*	*0.75*	*-*	*-*	*0.80*
09 Stipulation	Patients can write down what medical procedures they want to have done or not have done.					
	*0.77*	*-*	*0.78*	*-*	*-*	*0.82*
10 Decree	They are called durable powers of attorney.					
	*0.76*	*-*	*0.67*	*-*	*-*	*0.65*

Legend: Factor loadings show how strongly a variable is associated with an underlying factor. Loadings range from 0 to 1; loadings < 0.30 are considered weak; loadings between 0.30 and 0.50 are moderate, between 0.50 and 0.70 are strong, and loadings > 0.70 are considered very strong

**Fig. 2:**
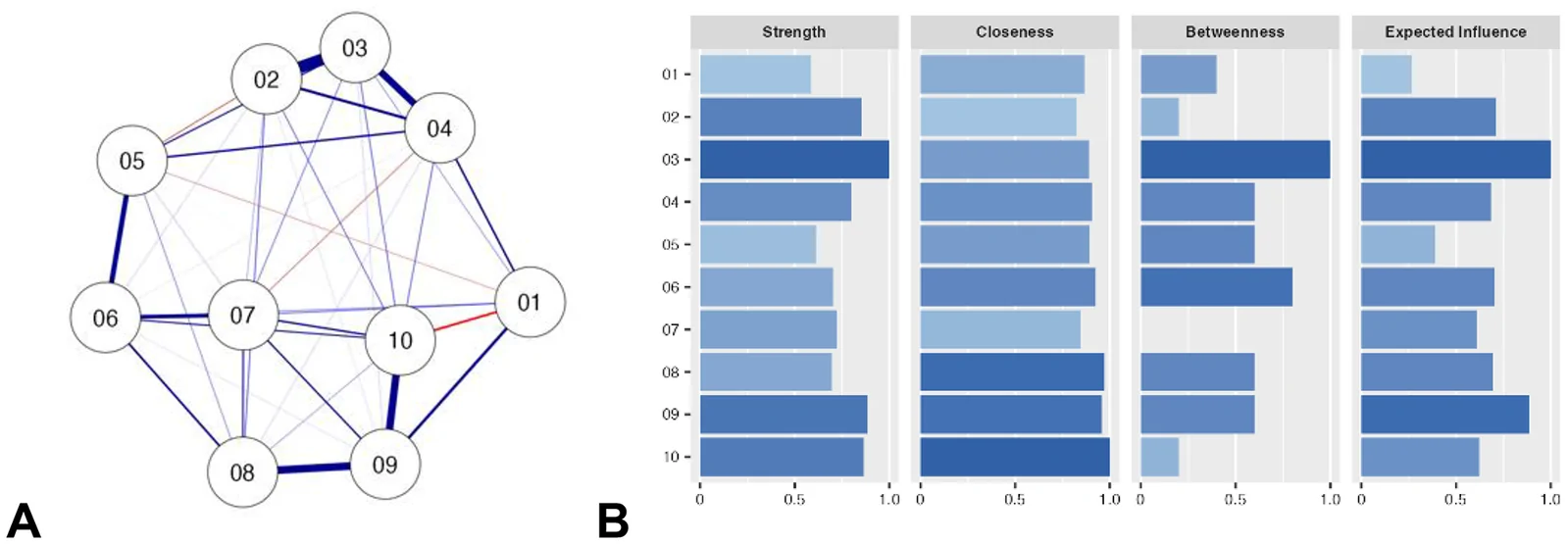
Network Model and Centrality Measures of the HCAT Questionnaire. Legend: **A**: Network Model of the HCAT-Questionnaire. **B**: Topographic characteristics of the network model. 01 Procedure; 02 Benefit; 03 Risk; 04 Alternative; 05 Shared Decision; 06 Deterioration; 07 Express; 08 Representative; 09 Stipulation; 10 Decree. Within the graphical representation, edges are the lines between the nodes (items) representing regularized partial correlations, which help estimate the relationship between two variables while controlling for all other variables. An edge indicates a dependent relationship between variables; the absence of an edge indicates that the variables are conditionally independent. The blue edges represent positive associations, while the red edges represent negative associations. The wider and more saturated an edge is represented, the stronger the association

The HCAT network (Fig. 2A) had an excellent stability index of 0.82. In the network representation, we can see two well-defined communities of nodes: one focused on the appreciation of a situation (i.e., risk, benefits, and alternatives), and the other on communicating a decision. The nodes had a mean strength of 0.77 (0.13), with nodes 03 (Risk) and 09 (Stipulation) having the highest strength indices within the network. The nodes had a mean closeness of 0.91 (0.06), with node 10 (decree) having closeness within the network. The nodes had a mean betweenness of 0.50 (0.30), with node 03 (Risk) having the highest betweenness within the network. The nodes had a mean expected influence of 0.66 (0.21), with nodes 03 (Risk) and 09 (Stipulation) having the greatest influence within the network (see Fig. 2B for further information).

### Comparison of the results of the models of the HCAT

For comparison of the Factorial Analysis and the Network Models, we used the chi-square test (χ²), the Comparative Fit Index (CFI), the Tucker‒Lewis Index (TLI), the Akaike Information Criterion (AIC), the Bayesian Information Criterion (BIC), and the Root Mean Square Error of Approximation (RMSEA) (for further details see Table 4). The chi-square test compares the observed data to the expected model, with smaller values suggesting a better fit. All of our models showed low *χ²* values. The CFI measures how well the proposed model fits the data compared to a baseline model. A CFI close to 1 (ideally above 0.95) indicates a good fit; the network model had the highest CFI, and the two- and three-factor models also met these criteria. The TLI evaluates the improvement in model fit relative to a null or independent model. A TLI value close to 1 (ideally above 0.95) suggests a good fit; only the network model fulfilled these criteria, although the two- and three-factor models were close. The AIC is a metric that balances model fit and complexity, thus penalizing excessive complexity to prevent overfitting. The BIC is a metric that penalizes model complexity more severely than the AIC, favoring simpler models and helping prevent overfitting. For both the AIC and BIC, the network model had the lowest values. The root mean square error of approximation (RMSEA) measures the discrepancy between the hypothesized model and the empirical data. An RMSEA of 0.06 or lower indicates a good fit; only the network model met this criterion [13, 20] (For further details, see Table 4).

**Table 4:** Fit indices for confirmatory factor analysis models

Fit Index	χ²	CFI	TLI	AIC	BIC	RMSEA
Cut-off Criteria	< 0.05	> 0.95	> 0.95	lowest	lowest	< 0.06
One Factor	*p*<.001	0.81	0.76	1705.73	1772.29	0.16
Two Factor	*p*<.001	0.96	0.94	1567.61	1637.50	0.08
Three Factor	*p*<.001	0.95	0.94	1568.27	1638.16	0.08
Network Model	*p*<.001	0.99	0.99	1071.53	1142.47	0.03

Legend: CFI: Comparative Fit Index; TLI: Tucker‒Lewis Index; AIC: Akaike Information Criterion; BIC: Bayesian Information Criterion; RMSEA: Root Mean Square Error of Approximation

## Discussion

In this study, we evaluated the administration and feasibility of the German version of the Hopkins Competency Assessment Tool (HCAT) [22, 23] in a clinical and a non-clinical population. In a second instance, we analyzed the psychometric properties of the HCAT, including the structure and underlying dynamics in a psychiatric sample. Overall, the findings were largely consistent with our expectations.

In our current trial, participants required approximately 10 min to hear, read, and answer each version of the HCAT, for a cumulative time of approximately 17 min. After two attempts (i.e., the HCAT 12th - and 8th -grade versions), most clinical participants completed the questionnaire. After a third attempt (i.e., the 6th -grade version), there was only a minority improvement. Non-clinical participants required less time to read the essay and answer the questionnaire; furthermore, they all flawlessly completed the assessment on the first attempt. These findings suggest that the instrument differentiates between individuals with presumed intact decisional capacity and those in clinical contexts where impairments may occur.

Compared with the original and previous HCAT studies [22, 23, 46], the administration procedures differed slightly: the present study separated the reading and listening phases rather than presenting both simultaneously. Nevertheless, administration times remained brief, supporting the feasibility of the HCAT as a structured screening instrument in clinical settings. Feasibility was further supported by the high proportion of participants who completed the assessment after the first or second administrations, whereas the additional benefit of a third administration appeared limited. Furthermore, all participants who initiated the screening also completed the procedure, even those who had to read the essay repeatedly and answer the questionnaire. Thus, showing through their engagement that the instrument is feasible [43]. Together, these findings suggest that the HCAT can be integrated into clinical assessment procedures without excessive administrative burden.

The internal consistency for those with two attempts was low (showing improvement in the response), while the internal consistency for those with three attempts was high (showing no improvement). This, in contrast to previous findings of HCAT, a learning effect could be shown beyond the second attempt [32]. The limited additional improvement observed beyond the second administration suggests that repeated exposure may provide diminishing returns in most cases. Within the clinical sample, performance varied depending on reading level and clinical context, supporting the view that decisional capacity is context-sensitive rather than a fixed trait. However, these findings are exploratory and should be interpreted cautiously due to small subgroup sizes.

In our sample, approximately 14% of patients did not meet the capacity threshold [22, 23], which is consistent with previous findings [33]. Despite the burden of illness, individuals with severe psychiatric disorders (such as schizophrenia or bipolar disorder) still have the capacity to make decisions about their medical treatment, and the proportion of individuals with no treatment decisional capacity is small [10]. Even those with a chronic course and severe symptoms can make treatment decisions after hospital admission [33]. However, we did not evaluate decisional capacity for a single, specific medical decision, which could be influenced by the complexity of the procedure and the circumstances [6, 7]. Thus, such an evaluation is out of the scope of the present analysis.

The German version of the HCAT demonstrated acceptable internal consistency and a coherent internal structure, supporting its reliability. Thus, corroborating previous results [41]. Both exploratory factor analysis and network modeling suggested that core components of decisional capacity—particularly understanding and risk–benefit appraisal—occupied central positions within the instrument’s structure. This pattern aligns with established theoretical models that emphasize comprehension, appreciation, reasoning, and the expression of choice as key elements of decisional capacity [5, 22, 23]. We believe that HCAT can provide insight into competency beyond mere understanding [41]. However, this assumption requires a comparative investigation with more sophisticated forms of competency assessment, especially those that consider the actual health and mental state [3, 4, 32, 41].

One strength of our study is the inclusion of a large clinical sample of psychiatric patients who were willing to participate, and the collection of data without prior selection during the screening process. Another strength is the inclusion of a non-clinical control group. One peculiarity of our sample is the recruitment of patients with schizophrenia, a chronic duration of illness, and a severe stage of the disorder. We selected this population because they are often excluded from clinical trials, because they might not be competent to give informed consent or be easily influenced [4].

The use of a network approach allows us to identify the dynamics among the elements of a construct [44]. In our case, the relationships among the several aspects of capacity assessment are beyond understanding [5]. From our perspective, this finding also reflects the relation between the elements of HCAT. We can infer that HCAT can quantify the essential elements of competency: understanding written and spoken language, extracting relevant information, and communicating a preference [5, 22, 23].

Our study has several limitations. First, the clinical characteristics of the sample, which consisted of patients with schizophrenia spectrum disorders, might limit the generalizability of our results to other diagnoses. However, the clinical characteristics of our sample (especially the HoNOS score) indicate a severe psychiatric disorder [14]. Thus, we can expect a better performance in the HCAT by other patients [4]. Our study was not primarily designed to translate, validate, and test the HCAT; it resulted from the necessity of a screening instrument to be able to assess consent capacity that does not focus on a specific medical decision [22, 23, 46] Apart from this, our study was conducted at a single study side, including participants and study personnel. To better validate the HCAT, multicenter studies should also be conducted. Where the influence of treatment setting, as well as a more detailed description of psychopathology and cognition, could be studied [33, 36].

Exploring or defining a cut-off for decisional capacity was not an objective of the present study. Although some patients performed poorly relative to others, scores were interpreted descriptively rather than as definitive determinations of capacity. Consistent with prior evidence, even individuals with severe psychiatric disorders often retain decision-making capacity, and the proportion of those lacking such capacity remains comparatively small [10, 33]. Accordingly, structured assessment tools such as the HCAT should complement, rather than replace, individualized clinical judgment.

As in the English version of the HCAT, we used a threshold of seven points to pass the HCAT, thereby improving comparability with previous reports [22, 23]. We could not determine cut-off points in our sample since we did not externally verify the capacity to consent, which would require further evaluation. Since we used no randomization, we cannot determine whether the difference is due to the more straightforward language (as used in the 8th - and 6th -grade versions) or to patients repeatedly receiving the information [11]. In contrast to the original publication, we decided not to read or listen to the essay simultaneously. We believe that simultaneous reading and listening do not align with clinical and study practice, in which patients provide written informed consent first, allowing them to read it [42]. Finally, the authors of the English version of the HCAT were not involved in the translation process; this version cannot be considered an official translation of the HCAT.

Another limitation of our study is the need for additional psychopathological or neurocognitive assessments further to differentiate the relationship between competency and other clinical parameters. Although such an assessment would be useful, it was not covered by the current ethics mandate, which allowed only screening procedures before providing informed consent for an ongoing clinical trial. Previous HCAT studies have shown no clear relationship between cognition and legal capacity [40]. In our study, we used the HoNOS score as a marker of severity and found no correlation with the HCAT score. However, it was assessed at the beginning of hospitalization, with a longer period for those in forensic treatment, so that an improvement could be expected [15].

In summary, the HCAT examines listening and reading comprehension, as well as the metacognitive and rational decision-making processes [4]. This is one, but not the only, basic condition of the capacity to decide. It also consists of understanding information relevant to a patient’s condition and the recommended treatment, reasoning about their potential risks and benefits, and expressing a choice [4, 5]. However, we did not evaluate how participants appreciate the nature of their situation or the consequences of their choices (as in forensic treatment) [29].

Finally, other aspects include the patients’ emotions and values. They later seem to increase their influence in more complex decisions [12, 19, 34]. The influence of circumstances and patients’ motivation should also be considered, as patients may be willing to make trade-offs to obtain a personal advantage (e.g., reimbursement). Furthermore, they might be subject to suggestions or even coercion (e.g., as in prison settings) [1, 29, 30]. Therefore, we should distinguish between “unreasonable” or “disadvantageous” decisions and the inability to make decisions with full respect for patients’ rights [34].

The findings of the present study may have implications for clinical and research practice beyond the field of psychiatry. Structured screening instruments appear useful for supporting the assessment of decisional capacity, particularly in situations in which clinical judgment alone may be difficult to standardize or document [4, 6, 34]. In addition, the HCAT may help investigators and clinicians optimize the language and structure of informed consent procedures by identifying information that requires particular emphasis or simplification [25]. This may become increasingly relevant in the context of digital or semi-automated informed consent procedures, which are gaining importance in clinical research and healthcare settings [18, 35]. Due to its general structure, the HCAT may provide a standardized framework for assessing core aspects of decisional capacity independent of a specific research question or clinical procedure [27, 45]. Such structured approaches may enhance transparency and patient safety, particularly among vulnerable populations, including psychiatric patients.

## Conclusions

The administration time for the HCAT was around 10 min; therefore, it may be suitable as a structured screening instrument in contexts where rapid assessment is required.

Psychiatric patients in general, but patients with schizophrenia, require easier language and more time to reflect on their consent. However, in most cases, they can give informed consent.

The instrument does not establish a categorical threshold for decisional capacity. Rather, it may help identify individuals who warrant a comprehensive evaluation.

Using a voice recording of the HCAT allows it to be administered by non-clinical staff or even automated.

## Electronic Supplementary Material

Below is the link to the electronic supplementary material.

Supplementary Material 1

## Data Availability

The data supporting this study’s findings are available from the corresponding author, SE, upon reasonable request.

## Abbreviations

AIC: Akaike Information Criterion
ANOVA: Analysis of Variance
BASEC: Business Administration System for Ethics Committees
BIC: Bayesian Information Criterion
CFA: Confirmatory Factor Analysis
CFI: Comparative Fit Index
EBIC: Extended Bayesian Information Criterion
EFA: Exploratory Factor Analysis
HCAT: Hopkins Competency Assessment Test
HoNOS: Health of the Nation Outcome Scale
ICC: Intraclass Correlation Coefficient
KMO: Kaiser–Meyer–Olkin
LASSO: Least Absolute Shrinkage and Selection Operator
MSA: Measure of Sampling Adequacy
RMSEA: Root Mean Square Error of Approximation
SD: Standard Deviation
SRMR: Standardized root mean square residual
TLI: Tucker‒Lewis Index
