# Supplementary material for: Translation and validation of a German version of the “Hopkins Competency Assessment Test,” a brief method for evaluating patients’ capacity to give consent
Source: BMC Psychiatry. 2026 Jul 24;26:811. doi: 10.1186/s12888-026-08401-6 (PMC13681955; doi:10.1186/s12888-026-08401-6)
Supplement: Supplementary file 1 [file 12888_2026_8401_MOESM1_ESM.docx]

**Translation and Validation of a German Version of the "Hopkins Competency Assessment Test," a Brief Method for Evaluating Patients’ Capacity to Give Consent.**

Hannah C. Keppeler, Mounira Jabat, Lena Machetanz, Mona Redlich Bossy, Achim Burrer, Stefan Vetter, Erich Seifritz, Johannes Kirchebner, Stephan T. Egger

**Appendix 01**

German Translation of the HCAT Essays

| **6. Klasse** | **8. Klasse** | **12. Klasse** |
| --- | --- | --- |
| Bevor ein Arzt einen Patienten behandeln kann, muss er ihm sagen, was er tun wird. Der Arzt erklärt dem Patienten auch, was bei der Behandlung gut laufen sollte, was bei der Behandlung schief gehen könnte und was der Arzt sonst stattdessen noch tun könnte.  Nachdem der Patient dies erfahren hat, kann er bestimmen, wie es weitergehen soll. Wenn er einverstanden ist, kann er dem Arzt sagen, er solle weitermachen. Wenn der Patient nicht einverstanden ist, sagt er dem Arzt, dass er das nicht möchte.  Manche Patienten sind sehr lange krank. Nach einer Weile kann es sein, dass sie wegen ihrer Krankheit nicht mehr so gut denken können. Dann verstehen sie die Erklärungen des Arztes vielleicht nicht recht und können auch nicht sagen, was dieser tun soll. Solche Patienten können vorher, solange sie noch gesund sind, ihrem Arzt sagen, mit welcher Person er dann sprechen soll. Sie müssen das auf Papier aufschreiben. Ein solches Papier nennt man *Patientenverfügung*.  In der Patientenverfügung steht, welche Person dem Arzt sagt, was er tun soll, falls der Patient dies wegen seiner Krankheit nicht selbst sagen kann. In der Patientenverfügung steht auch, welche Behandlungen der Patient möchte und welche er nicht möchte, wenn er krank wird. | Bevor ein Patient eine medizinische Behandlung erhält, muss der Arzt ihn über den Ablauf der Behandlung informieren und vor allem Folgendes genau erklären: was bei der Behandlung gut laufen sollte, was bei der Behandlung schief gehen könnte und was der Arzt anstelle der vorgeschlagenen Behandlung sonst noch tun könnte.  Nach dieser Information kann der Patient entscheiden, ob er mit der medizinischen Behandlung einverstanden ist oder nicht.  Patienten, die eine lange Zeit krank sind, können eventuell als Folge ihrer Krankheit nicht mehr genau verstehen, was ihnen der Arzt erklärt. Wenn das passiert, sind manche Patienten nicht mehr in der Lage, sich für oder gegen eine spezifische medizinische Behandlung zu entscheiden. Dann muss jemand anders für sie entscheiden und das den Ärzten mitteilen. Die betroffenen Patienten können deshalb – solange sie noch gesund sind – ihrem Arzt sagen, was getan werden soll, falls sie einmal nicht mehr selbst entscheiden können. Sie können auch eine Person bestimmen, die dann mit dem Arzt spricht. Das alles müssen die betroffenen Patienten auf Papier schriftlich festhalten. Ein solches Papier nennt man *Patientenverfügung*.  In der Patientenverfügung steht, welche Person medizinische Entscheidungen treffen soll, falls die Patienten dazu nicht mehr in der Lage sind. Die Patienten können auch allgemein aufschreiben, was nach ihren Wünschen getan werden soll, wenn sie nicht mehr fähig sind selbst zu entscheiden. | Bevor sich ein Patient einer medizinischen Behandlung unterzieht, muss er vom Arzt über die betreffende Behandlung informiert werden.  Der Patient sollte verstehen, welche Vorteile von der Behandlung zu erwarten sind, welche Risiken zur Behandlung bekannt sind und welche eventuellen Alternativen zur vorgeschlagenen Behandlung bestehen.  Ein Patient, der diese Informationen zur Kenntnis nimmt, hat die Möglichkeit der Behandlung zuzustimmen oder sie abzulehnen.  Patienten mit einer chronischen Erkrankung können die Fähigkeit verlieren, die Informationen des Arztes zu verstehen. Für sie wird es deshalb schwierig, eine verantwortungsvolle Entscheidung zu ihrer eigenen Gesundheitsversorgung zu treffen. In diesem Fall muss die Entscheidungsbefugnis an jemanden delegiert werden. Solche Patienten können im Voraus rechtlich-formelle Vorgaben hinterlassen und schriftlich festhalten, was sie als medizinische Behandlung wünschen und welche Person weitere Entscheidungen treffen soll. Solche schriftlich festgehaltenen Vorgaben werden *Patientenverfügung* genannt.  In der Patientenverfügung können Patienten nicht nur bestimmen, wer die medizinischen Entscheidungen für sie treffen darf, sondern auch, welche Beschränkungen dieser Entscheidungsinstanz auferlegt werden sollen. |

German Translation of the HCAT Questionnaire

| Welche sind die vier Dinge, die ein Arzt einem Patienten erzählen muss, bevor er eine Behandlung beginnt? | |
| --- | --- |
| Item 01  Procedure | Was der Arzt tun wird. |
| Item 02  Benefit | Was gut laufen könnte. |
| Item 03  Risk | Was schieflaufen könnte. |
| Item 04  Alternative | Was der Arzt stattdessen tun könnte. |
| Richtig oder Falsch? | |
| Item 05  Shared Decision | Nachdem der Patient über die Behandlung aufgeklärt worden ist, kann er entscheiden, die Behandlung nicht zu machen. |
| Was kann manchmal mit dem Denken eines Patienten passieren, der für eine lange Zeit krank ist? | |
| Item 06  Deterioration | Nach einer Zeit könnte es sein, dass das Denken des Patienten nicht mehr so gut ist wie jetzt. |
| Vervollständigen Sie den Satz: Ein Patient, dessen Denken schlecht geworden ist, könnte nicht fähig sein... | |
| Item 07  Express | dem Arzt zu sagen, was er möchte. |
| Welche beiden Dinge sollten solche Patienten ihrem Arzt und ihrer Familie sagen, bevor ihr Denken schlechter wird? | |
| Item 08 Representative | Patienten können aufschreiben, mit wem der Arzt an ihrer Stelle sprechen könnte, damit derjenige medizinische Entscheidungen für sie trifft. |
| Item 09  Stipulation | Patienten können aufschreiben, welche medizinische Behandlungen sie möchten und welche sie nicht möchten. |
| Wie nennt man solche Anweisungen für Ärzte und Familienmitglieder? | |
| Item 10  Decree | Patientenverfügung. |
